# Case Report: Importance of high-throughput genetic investigations in the differential diagnosis of unexplained erythrocytosis

**DOI:** 10.3389/pore.2025.1612037

**Published:** 2025-03-10

**Authors:** Zsófia Flóra Nagy, György Pfliegler, János Kósa, Kristóf Árvai, Ildikó Istenes, Attila Doros, Botond Timár, Péter Lakatos, Judit Demeter

**Affiliations:** ^1^ Department of Internal Medicine and Oncology, Semmelweis University, Budapest, Hungary; ^2^ Centre of Rare Diseases, Faculty of Medicine, University of Debrecen, Debrecen, Hungary; ^3^ Department of Interventional Radiology, Heart and Vascular Centre, Semmelweis University, Budapest, Hungary; ^4^ 1st Department of Pathology and Experimental Cancer Research, Semmelweis University, Budapest, Hungary

**Keywords:** polyglobulia, WES, diagnostic, chuvash polycythaemia, pyruvate kinase hyperactivity

## Abstract

**Introduction:**

Polycythemia indicates the pathological increase in the number of red blood cells and the rise of hematocrit values. Polyglobulia can be of primary or secondary origin, with the most common primary polycythemia being a myeloproliferative neoplasm, polycythemia vera. Polyglobulia patients may develop cardiovascular complications and thromboembolic events. The gold standard of first-line treatment in polycythemia vera is phlebotomy, which is indicated to keep the hematocrit value below 0.45. Until now the goal to be achieved in secondary polyglobulia has been similar. In secondary polyglobulia this rule of thumb needs to be re-evaluated as shown by the example of two patients suffering from different rare, genetically determined polyglobulias. In our article we present the case of these two patients and discuss the diagnostic and therapeutic principles to be applied in patients with rare, genetically determined polyglobulias.

**Patients and methods:**

After completing the usual diagnostic algorithm for polyglobulia no cause could be identified in two of our male patients. Therefore, we set out to perform whole exome sequencing in both patients. Our analysis did not include copy number analysis.

**Results:**

In Patient 1 the p.Ser179Pro variant in the *VHL* gene was detected in the homozygous state, which is classified as likely pathogenic according to the ACMG guidelines. Homozygous *VHL* mutations are implicated in Chuvash polycythemia. Segregation analysis was declined by the family. In Patient 2 the *PKLR* gene p.His306Gln variant was detected in the heterozygous form. The gene plays a role in pyruvate metabolism. Family screening did not detect this variant in healthy family members.

**Discussion:**

We identified rare, possibly pathogenic genetic variants in two patients with polyglobulia and as a consequence of the genetic diagnosis we implemented individualized patient monitoring. We recommend the utilization of high-throughput genomic testing in cases with unexplained polyglobulia.

## Introduction

According to the definition of polycythemia by the World Health Organization hemoglobin levels of >165 g/L in men or >160 g/L in women and hematocrit values >49% in men or >48% in women are considered pathological [1]. Primary causes of polycythemia are mutations in genes involved in the regulation of erythropoiesis, while secondary polycythemia may develop as a consequence of chronic cardiopulmonary disease or an erythropoietin-secreting process [2, 3]. The most common primary polycythemic disease is polycythemia vera (PV) which has an incidence of 1.52/100′000/year in the Hungarian region of Szabolcs-Szatmár-Bereg based on 36 years of data collection. Interestingly, the incidence of PV is increasing by 0, 3 persons/year [4].

When evaluating a patient with polyglobulia a thorough medical history (focusing on hyperviscosity symptoms, thrombotic events, aquagenic pruritus, erythromelalgia) and a detailed physical examination are of utmost importance. A complete blood count and blood smear examination should be part of the laboratory work-up. Measurement of erythropoietin (EPO) levels can differentiate between primary (low or normal EPO levels suggest a physiological response to hypoxia) and secondary (elevated EPO levels may reflect a disrupted reaction to hypoxia) causes of polyglobulia. Hemoglobinopathies especially those with high oxygen binding affinity must also be excluded. Testing for the most common chronic myeloproliferative neoplasm-associated mutations, such as *Janus kinase 2* (*JAK2), Calreticulin (CALR), Myeloproliferative leukemia virus oncogene (MPL)* genes*,* and performing a bone marrow biopsy may also aid in the diagnostic process [5].

In our research we set out to investigate the genetic background of two patients with polycythemia of unknown origin utilizing next-generation sequencing technologies. Both patients had previously undergone extensive testing to uncover the cause of their severe polycythemia but no diagnosis had been made.

### Patient #1

Patient #1 is a male patient born in 1988. Upon his birth his unusually plethoric skin color was noted. At birth, his Hgb level was 193 g/L, and at 3 months of age his Hgb level dropped to only 152 g/L (normal range: 90–141 g/L). He reported no relevant family history of hematological diseases.

At the age of 9 years, during a routine checkup, he was diagnosed with polyglobulia (Hgb 212 g/L Hct: 64%). From the age of 11 years, regular phlebotomies were performed. His polyglobulia persisted despite the regular phlebotomies and from the age of 28 years a slightly enlarged spleen was consistently noted on both physical examinations and abdominal ultrasound exams as well. From this time on a constantly rising EPO level was also detected.

The patient was examined for possible causes of polyglobulia: an echocardiogram did not reveal any congenital heart disease or septal defects, and hemoglobin electrophoresis did not confirm the presence of hemoglobinopathy. A bone marrow examination was also been performed at the age of 9 years, but the records regarding this examination are not available. Several imaging studies were performed to rule out malignancy. Due to the constantly rising EPO levels, selective vein catheterization was performed and elevated EPO levels were noted in samples taken from the superior mesenteric vein, the splenic vein and the hepatic portal vein. The measured EPO levels were around 40 mU/mL in all examined veins.

At the age of 33 years, a PET-CT examination was performed which did not reveal any malignancy or suspicious FDG uptake.

Molecular genetic testing for *JAK2, CALR*, *MPL* and *Hemoglobin beta locus (HBB)* genes was also conducted with negative results.

**FIGURE 1:**
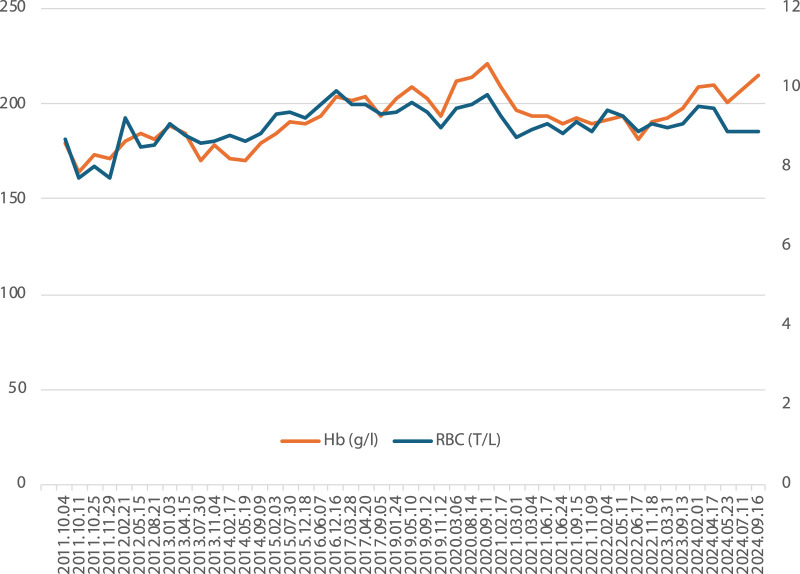
Hemoglobin and red blood cell counts of Patient #1 over 13 years of follow-up.

The patient attends regular check-ups and does not report any symptoms of hyperviscosity. Figure 1 shows the hemoglobin and hematocrit values of Patient #1 over our follow-up. He does not take any medication regularly.

### Patient #2

Patient #2 is a male patient born in 1963. He presented to a hematologist’s office at the age of 35 years after a routine blood count examination revealed his polyglobulia (at diagnosis Hgb: 224 g/L, Hct 70%). His blood examinations showed hemolytic signs with regenerative properties (elevated reticulocyte count, elevated indirect bilirubin, elevated LDH). EPO levels were within the normal range at each measurement and hypoxia-induced factor 1 α levels were also within the normal range. His past medical history included varicose veins in both calves and a varicectomy. At the age of 51 years, several kidney cysts were detected, raising the suspicion of polycystic kidney disease, which was later excluded by next-generation sequencing genetic testing: negative for *Polycystin 1* (*PKD1), Polycystin 2 (PKD2)*, and *Ciliary IPT domain-containing fibrocystin (PKHD1)* genes. At the age of 60 years he was diagnosed with cholelithiasis after an episode of cholecystitis. Cholecystectomy is scheduled in the near future. His family history was negative for hematological diseases.

*JAK2, Von Hippel-Lindau tumor suppressor (VHL), Erythropoietin receptor (EPOR), HBB, Cystic fibrosis transmembrane conductance regulator (CFTR)* and *Endoglin* (*ENG)* genes were sequenced with negative results. Hemoglobin electrophoresis was also performed and revealed no hemoglobinopathies. He also underwent an iliac crest biopsy which was reported to be negative.

He is followed up in our department and does not report any symptoms of hyperviscosity. He regularly takes beta-blockers for his hypertension and allopurinol for elevated uric acid levels. He was first prescribed a platelet aggregation inhibitor (100 mg acetylsalicylic acid/day) in 2003. He was taking the prescribed medication on and off, but it was brought to his attention again and he was strongly advised to take the acetylsalicylic acid.

## Methods

Both patients gave their informed consent to the examinations and underwent genetic counseling prior to genetic testing. Post-test genetic counseling and family testing were also offered to our patients. This study was conducted in accordance with the Helsinki Declaration and its amendments and was approved by the Medical Research Council’s Ethical Committee (BMEÜ/2864-1/2022/EKU).

Whole exome sequencing was performed in both patients to uncover the genetic background of polycythemia. Genomic DNA was extracted from EDTA-containing blood samples with a commercially available kit according to the manufacturer’s recommendations. For target enrichment, a Twist Exome 2.0 kit was used and next-generation sequencing was completed on an Illumina NovaSeq sequencing device. Variant calling was performed on the Illumina Dragen server.

In the case of Patient #1 the average variant depth was 132, and 20x coverage was achieved on 98.4% of reads. The Ti/Tv ratio of variant calling accuracy was 2.95, while 98% of variants had a Franklin quality score above 40, indicating that the probability of an incorrect base call is 1 in 10,000. The same metrics for Patient #2 were as follows: average variant depth was 111, and 20x coverage was achieved on 98.9% of reads. The Ti/Tv ratio of variant calling accuracy was 3.01% and 97% of variants had a quality score above 40.

The variant interpretation was performed according to the 2015 guidelines of the American College of Medical Genetics and Genomics (ACMG) [6]. For variant prioritization online variant assessment platforms, such as Varsome and Franklin^1^ were implemented [7]. First, we followed a phenotype-driven data analysis and applied a virtual filtering panel. We used the following Human Phenotype Ontologies (HPOs): HP:0001901, HP:0001900, HP:0001898, HP:0001871, and HP:0020054. As a second approach, we screened for variants already submitted to ClinVar as pathogenic or likely pathogenic. Finally, we also checked the ACMG Secondary Findings V3.2 gene set.

## Results

### Patient #1

In Patient #1 the *VHL*(NM_001354723.2):c.535T>C; p.Ser179Pro variant was detected in the homozygous form. According to the current ACMG classification, it is likely to be pathogenic. The variant fulfills the PM2 pathogenic moderate (allele frequency in healthy non-Finnish Europeans is 0.0015%), PP2 pathogenic supporting and PP5 pathogenic strong criteria of the ACMG classification system. The variant has been submitted to ClinVar as pathogenic in association with Chuvash polycythemia (RCV001007626). We do not have further information on the genetic inheritance in case of our patient, as his parents were not available for segregation analysis. Based on the molecular genetic testing we diagnosed the patient with Chuvash polycythemia.

Following the diagnosis of Patient #1 with Chuvash polycythemia, extensive cardiological and pulmonological examinations were performed to assess the possible involvement of the pulmonary circulation and to screen for the presence of pulmonary hypertension. The patient’s estimated pulmonary pressure was within the normal range and no pathological signs were noted on echocardiography. Spirometry showed minimal obstructive changes in the smaller airways and an elevated residual volume (128%). However, arterial blood gas examination and pulmonary diffusion tests showed physiological results. We plan to continue our patient’s regular cardiological and pulmonological examinations to detect potential pulmonary involvement as early as possible. After establishing the diagnosis we started platelet aggregation inhibitor (100 mg acetylsalicylic acid/day) as a preventive measure.

### Patient #2

Next-generation sequencing identified the *pyruvate kinase* (*PKLR)* (NM_000298.6) c.918C>A, p.His306Gln variant in the heterozygous form in Patient #2. The variant is currently classified as a variant of uncertain significance (VUS) leaning pathogenic, as it fulfills the PM1 pathogenic moderate, the PP2 pathogenic supporting and the PM2 pathogenic moderate criteria of the ACMG classification system. The variant has a low allele frequency in non-Finnish Europeans (0.0103%) and several prediction tools suggest its deleterious effect (GenoCanyon, fitCons, MetaLR, FATHMM). The variant has one submission in ClinVar as a VUS (RCV000369340). Segregation analysis of the patient’s asymptomatic mother and asymptomatic daughter did not identify the variant in the asymptomatic family members.

The gene is associated with pyruvate metabolism disorders such as red blood cell pyruvate kinase deficiency (autosomal recessive inheritance) and pyruvate kinase hyperactivity (autosomal dominant inheritance).

## Discussion

### Patient #1

To our knowledge, this is the first report of a Hungarian patient with Chuvash polycythemia. *VHL* variants were reported to be sporadic in congenital polyglobulia patients with elevated EPO levels of Italian origin in a study from 2005 [8]. In a cohort of 34 congenital polyglobulia patients only 4 were found to be homozygous (4/34, 11.8%) for a pathogenic *VHL* mutation. However, the exact prevalence of *VHL* mutations in congenital polycythemic patients is unknown due to the limited cohort size [9]. To date, only two homozygous *VHL* mutations presenting as congenital polyglobulia phenotype have been described, the Chuvashia founder mutation, p.Arg200Trp and the Croatian mutation, p.His191Asp [10, 11]. The variant reported by our group is the third *VHL* mutation identified in the homozygous state in a patient with severe polyglobulia.

*VHL* single nucleotide polymorphism (rs779805)-associated polycythemia was also observed in a cohort of Hungarian patients followed up for polyglobulia of unknown cause. GG homozygotes and GA heterozygotes represented 66% of the investigated population which was significantly higher than in the control group. Interestingly, an abundance of tumors was observed in the relatives of the studied probands. However, either tumors (clear cell renal cancer, colon cancer, melanoma, nodular sclerosis Hodgkin lymphoma, non-Hodgkin lymphoma, bony tumors, gynecological cancers, and hemangioma) or polycythemia were present in the subjects, but never both [12].

The phenotype of patients homozygous for the two previously described homozygous variants (p.Arg200Trp and p.His191Asp) is consistent with the phenotype of our patient. However, the EPO levels, mean corpuscular volume, mean corpuscular hemoglobin levels, and mean corpuscular hemoglobin concentrations observed in our patient were closer to those of patients with the p.Arg200Trp mutation [13]. To better understand this phenomenon, further research is needed to elucidate the molecular properties of the *VHL* p.Ser179Pro variant.

Chuvash polycythemia has characteristics of both primary and secondary polycythemic diseases, as the erythroid progenitors of these patients show EPO hypersensitivity and EPO levels are elevated in Chuvash polycythemia [14]. The secondary increase in EPO levels can be explained by the fact that all Chuvash polycythemia-associated *VHL* gene variants have been shown to exhibit decreased protein stability and reduced binding of the Hypoxia-inducible factor 1 (HIF1) protein, thereby disturbing the physiological sensing of cellular hypoxia [15, 16]. The link between the primary and secondary erythrocytic processes was proposed recently by a Canadian group of researchers. They found that Chuvash polycythemia-associated *VHL* variants do not cause the ubiquitin-mediated breakdown of the activated JAK2 protein. In a mouse model of Chuvash polycythemia caused by the *VHL* p.Arg200Trp mutation, an investigational JAK2 inhibitor molecule was able to salvage the phenotype of the mice. Based on these findings highly selective JAK2 inhibitors may represent a promising future therapeutic option for patients with this disease [17].

In terms of tumorigenesis, we confirm the results of other groups: we did not detect any malignancy in our patient by the age of 36 years. Since VHL-associated tumors have an age-dependent but generally high penetrance, we hypothesize similar to other research groups that biallelic *VHL* mutations do not confer an increased risk for tumors [16, 18]. All reported *VHL* variants associated with Chuvash polycythemia and lack of malignancy are located at the 3′ end of the gene (both in exon 2 and exon 3), as is our reported p.Ser179Pro variant. Our variant is the third reported Chuvash polycythemia-associated *VHL* gene variant that does not promote tumorigenesis [13, 16].

Chuvash polycythemia is associated with an increased risk of cerebral and non-cerebral vascular events and elevated pulmonary tension [19]. In a case report of a Bangladeshi infant carrying the p.Asp126Asn homozygous variant in the *VHL* gene severe pulmonary hypertension developed at 16 months of age and the patient died at 2 years of age due to the complications of a viral infection [20]. Our patient tested negative for pulmonary hypertension at the age of 35 years, thus we consider the p.Ser179Pro variant to be associated with a more benign course of the disease. However, primary prevention against stroke and thrombosis is recommended.

### Patient #2

When discussing the pathogenesis of polyglobulia in this patient it is interesting to note that in 1965 a 6-7-fold increased intraerythrocytic adenosine triphosphate (ATP) level was reported when examining a patient diagnosed with glucose-6-phosphate dehydrogenase deficiency [21]. Furthermore, the trait was found to be inherited in a dominant manner and the condition was labeled as “hereditary high ATP content of the erythrocytes” [21]. Pyruvate kinase hyperactivity as an entity was first reported in 1980 based on the examination of 4 family members who all presented with polycythemia and were later tested for pyruvate kinase activity which was found to be almost four times higher than that of control patients. Further investigations revealed elevated intraerythrocytic ATP levels and lower levels of 2,3-diphosphoglycerate [22]. The first variant of the *PKLR* gene to be detected in pyruvate kinase hyperactivity was p.Gly37Glu [23]. To date only 3 families with pyruvate kinase hyperactivity have been identified, and our patient is in the fourth reported family [22, 24].

Pyruvate kinase deficiency must not be confused with pyruvate kinase hyperactivity. Homozygous or compound heterozygous variants of the *PKLR* gene cause pyruvate kinase deficiency or congenital non-spherocytic hemolytic anemia-2, which is the most common hereditary glycolytic enzyme disorder [25]. The phenotype associated with pyruvate kinase deficiency is moderate to severe, often transfusion-dependent hemolytic anemia, splenomegaly and increased osmotic fragility of the red blood cells [26].

Pyruvate kinase hyperactivity is most often associated with mild polycythemia and shortened red blood cell lifespan. In contrast to pyruvate kinase deficiency, red blood cell morphology in pyruvate kinase hyperactivity is unaltered [23]. In our patient the polycythemia is more severe than mild which can be attributed to an even higher enzymatic activity of the pyruvate kinase protein. Unfortunately, this enzymatic activity assay is not routinely available in our country, thus we cannot accurately determine this property. It would be of utmost interest to determine the underlying pathomechanism as it was different in all three families (altered enzyme kinetic properties, persistent expression of an isoform and increased expression of a kinetically normal enzyme isoform) [22, 24]. In a recent publication it was proposed that the *PKLR* gene and its expression are under the regulation of an as yet unknown genetic factor; therefore, more research is needed to characterize the nature of this disease. However, the delineation of the disease is limited by the ultra-rare nature of the condition [24].

## Conclusion

Our results show that high-throughput genetic testing may be important in the diagnostic workup of polyglobulia after more common causes of the disease have been ruled out. Our findings also highlight that genetic data may influence patient management and thus may help to establish individualized treatment plans.

## Data Availability

The original contributions presented in the study are included in the article/supplementary material, further inquiries can be directed to the corresponding author.

## Footnotes

1
https://franklin.genoox.com
